# Effect of solution focused approach on women aged 35 or over with in vitro fertilization-embryo transfer: A quasi-experimental trial

**DOI:** 10.1371/journal.pone.0316771

**Published:** 2025-03-19

**Authors:** Nan Tang, Lei Xie, Mengyue Pei, Jing Wang, Junping Hu, Yuan Gao

**Affiliations:** 1 Department of Nursing, the First Medical Centre of PLA General Hospital, Beijing, China; 2 School of Nursing, Lanzhou University, Lanzhou, China; 3 Renji Hospital, Shanghai Jiaotong University, Shanghai, China; 4 Henan Kaifeng College of Science Technology and Communication, Kaifei, Henan, China; 5 Department of Gynaecology and Obstetrics, Chinese PLA General Hospital, Beijing, China; 6 The first hospital of Lanzhou University, Lanzhou, Gansu, China; University of Rijeka Faculty of Medicine: Sveuciliste u Rijeci Medicinski fakultet, CROATIA

## Abstract

**Background:**

We aimed to explore the influence of solution-focused approach (SFA) on anxiety and depression, sleep quality, quality of life and clinical pregnancy rate among women aged 35 or over undergoing in vitro fertilization-embryo transfer (IVF-ET).

**Methods:**

The study was performed at the reproductive center in a public hospital in Lanzhou city. Totally, 112 women were enrolled in this study, and were divided into group SFA (n = 56) and group control (n = 56). The patients in the group SFA completed five sessions (30 minutes/turn), and patients in the group control received routine care.

**Results:**

The intervention group showed a significant decrease in anxiety (t =  11.906, P <  0.001) and depression scores (t =  14.991, P <  0.001), as well as PSQI scores (t =  7.055, P <  0.001), and increased FertiQoL scores (t =  -2.828, P <  0.001). Comparing the two groups after the intervention, the intervention group demonstrated significantly lower SAS scores (t =  -10.348, P <  0.001), SDS scores (t =  -8.416, P <  0.001), and PSQI scores (t =  -5.087, P <  0.001), while FertiQoL scores were higher than the control group (t =  2.389, P =  0.019). The intervention group reported a satisfaction rate of 96.2% to 100% with the SFA.

**Conclusions:**

SFA can help relieve anxiety and depression, improve sleep quality and reproductive life quality. Improvement in psychological distress might not contribute to increasing female fecundity. Patients in the group SFA were satisfied with the intervention.

**Trial registration:**

Chinese Clinical Trial Registry (ChiCTR2300075444).

## Introduction

Infertility refers to the condition where individuals have regular sexual activity without using contraception for at least one year but have not achieved pregnancy [1]. The prevalence of infertility in women of childbearing age is 3.5% to 16.7% in developed countries and 6.9% to 9.3% in developing countries [2]. In China, the prevalence of infertility ranges from 10.5% to 26.9% [3]. The International Federation of Gynecology and Obstetrics (FIGO) defined women aged 35 years and above as “advanced maternal age” in 1958 [4]. Compared to non-advanced maternal age, these women have poorer pregnancy and delivery outcomes, higher fetal mortality rates, and increased maternal complications [5]. According to the “Clinical Practice Guidelines for Assisted Reproductive Technology in Advanced Maternal Age Women in China,” the age of 35 years is considered as the dividing line for advanced maternal age in reproductive health for women. As women age, their reproductive risks increase, fertility declines, and the risks of infertility and miscarriage rise [6]. In this study, we adopted the age of 35 years as the cutoff value for defining advanced maternal age in women with infertility.

The success rate of Assisted Reproductive Technology (ART) cycles decreases with increasing maternal age [5]. Many patients have high expectations during treatment, which may lead to negative emotions such as anxiety and depression [7]. Previous studies have shown that negative emotions in infertility patients can lower pregnancy rates and also impact their family relationships and quality of life [8]. In the field of assisted reproductive technology, age is an important indicator for predicting the outcome of IVF-ET treatment [9]. Advanced maternal age infertility patients often exhibit reduced responsiveness to ovulation-inducing medications, resulting in fewer retrieved eggs and higher cycle failure rates, which poses a challenge of low success rates and higher fertility risks [5]. Research indicates that as age increases, the severity of anxiety and depression in infertility patients also tends to worsen [10].

Negative emotions primarily affect the body’s endocrine system through the Hypothalamic-Pituitary-Adrenal axis (HPA), leading to increased cortisol secretion, which, in turn, influences the Hypothalamus-Pituitary-Gonad Axis (HPGA) and alters hormone secretion, impacting endometrial receptivity and uterine blood flow, thereby affecting treatment outcomes [11]. Alongside the progressively increased age of infertile couples, environmental and behavioural factors, including non-optimal lifestyle habits, should be considered. Among these, sleep disorders have been suggested to be linked to human fertility [12]. The incidence of sleep disorders in patients receiving IVF-ET treatment ranges from 46% to 57.1%, higher than that in normal adults (14% to 40%) [13]. Sleep quality can affect the quality of oocytes and embryos, thus having adverse effects on pregnancy outcomes [14]. The prevalence of reduced ovarian reserve is about 2.489 times higher in women who experience sleep disorders during treatment, especially in those aged 35 and over [15]. Moreover, poor sleep quality and decreased quality of life in IVF-ET patients can, in turn, impact their psychological state and increase negative emotions [16].

Therefore, the negative emotions, such as anxiety and depression, that may exist in advanced maternal age infertility patients could further impact their quality of life and sleep quality, and even have adverse effects on pregnancy outcomes and fetal development. Effective psychological intervention can improve patients’ anxiety, depression, and sleep quality, thereby enhancing their quality of life [17,18]. Therefore, this study aims to apply the Solution Focused Approach (SFA) to women with advanced maternal age undergoing IVF-ET, with the goal of alleviating negative emotions such as anxiety and depression, improving sleep quality and reproductive quality of life, and positively influencing pregnancy outcomes.

The SFA also known as the focus on solutions model, emphasizes that individuals are the experts of their own lives. The core of the intervention is to focus on positive behaviors, explore individual’s inner potential, and promote holistic personal development to achieve desired outcomes [19]. Currently, SFA has been applied in clinical nursing, aiming to assist patients in utilizing their strengths, potentials, and available resources to find solutions to problems rather than focusing on the origins of the problems [20]. The SFA is a standardized psychological intervention with five stages, including problem description, goal setting, exploring exceptions, providing feedback, and evaluating progress [21]. The implementation principle is patient-centered, focusing on eliciting patients’ potentials and encouraging active participation to mobilize their resources and strengths, ultimately achieving the goal of recovery [22]. This approach has shown significant effectiveness in various populations, effectively alleviating negative emotions, and improving self-efficacy and health behaviors [22–24]. Therefore, this study aims to apply SFA in clinical intervention for women with advanced maternal age undergoing IVF-ET to explore its effects on improving their psychological status, sleep, and quality of life. Unlike previous studies that largely focused on younger populations or more general psychological interventions, this study uniquely tailors SFA to address the needs of women experiencing age-related infertility challenges.

## Materials and methods

### Study design and participants

The study was retrospectively registered as a clinical trial (ChiCTR2300075444; Date of registration: 05/09/2023), and approval was obtained from the medical ethics committee of School of nursing, Lanzhou University. Due to the COVID-19, many administrative institutions were suspended. The clinical trial application cannot be approved, therefore we are unable to complete the prospective trial registration. The Our study adheres to CONSORT guidelines [25]. This was a single-center, quasi-randomized controlled trial, to assess the effects of SFA interventions on primary and secondary outcomes. The primary outcomes were depression and anxiety. The secondary outcomes were sleep quality, reproductive life quality, and clinical pregnancy rate.

### Participants

The enrolled women were recruited at a Reproductive Medicine Special Hospital from December 2021 to December 2022. The study was approved by the ethics committee of the Nursing School, Lanzhou University, Lanzhou, China (LZUHLXY20210053). This study complied with the Declaration of Helsinki. Written informed consent was obtained from all study participants. Inclusion criteria for the study were as follows: [1] Patients who underwent IVF-ET fresh cycle transplantation; [2] Female patients aged ≥ 35 years; [3] Ability to independently complete various questionnaires and scales used in the study; [4] Written informed consent and voluntary participation in the research; [5] Self-rating anxiety scale score ≥ 50 (standard score) and self-rating depression scale score ≥ 53 (standard score). Exclusion criteria were as follows: [1] Severe acute or chronic illness; [2] Male partners with severe oligozoospermia; [3] Patients currently participating in similar research; [4] Patients who received psychological treatment in the mental health department within the past two months; [5] Self-rating anxiety scale score > 70 or self-rating depression scale score > 72 (they were advised to visit the mental health unit for further assessment and treatment).

### Procedures

A total of 112 women were recruited for this study. Recruitment and evaluation of participants were conducted by a trained nurse to ensure consistency and reliability. Participant information was kept strictly confidential and anonymous throughout the study. Based on the treatment card tail numbers, participants were randomly assigned to the control group (n = 56) or the SFA (Solution-Focused Approach) group (n = 56). On the first day of treatment initiation (T1), the researchers provided detailed explanations to participants regarding the study’s purpose, research process, precautions, and anticipated outcomes.

**Fig 1:**
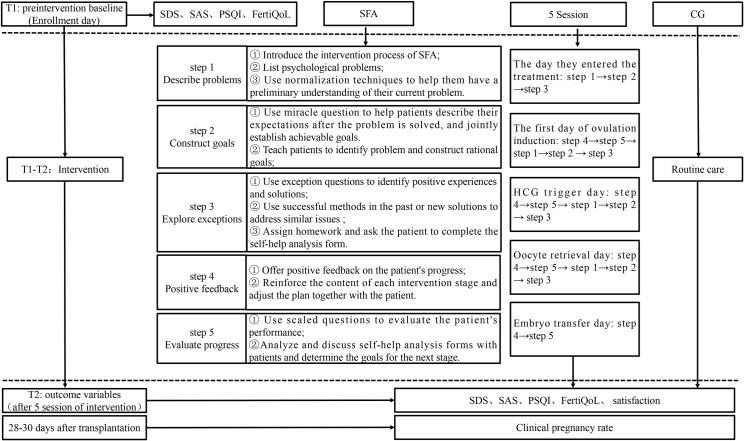
Intervention process of two groups. SAS, self-rating anxiety scale; SDS, self-rating depression scale; PSQI, Pittsburgh sleep quality index scale; FertiQoL, Chinese version of reproductive quality of life scale; SFA, solution-focused approach; CG, control group.

At T1, participants completed baseline self-reported questionnaires, which gathered data on demographics, clinical characteristics, and outcome variables, including depression, anxiety, sleep quality, and reproductive quality of life. Participants in the SFA group received their first intervention session immediately following the T1 assessment. Subsequent evaluations (T2) were conducted post-intervention on the day of embryo transfer. At T2, participants completed questionnaires assessing depression, anxiety, sleep quality, reproductive quality of life, and satisfaction with the intervention. Additionally, clinical pregnancy rates were recorded 28–30 days post-embryo transfer. A detailed study protocol and assessment timeline are illustrated in Fig 1.

### Interventions

Participants in the control group received routine nursing care, which included the following components: [1] Day of Treatment Initiation: Introduction to in vitro fertilization and embryo transfer (IVF-ET) procedures, treatment plans, medication guidelines, and precautions. [2] Ovulation Induction Period: Explanation of the purpose, principles, and medication requirements for ovulation induction. Participants received an assisted conception manual and a scheduled follow-up date. They were advised to maintain a balanced diet, engage in moderate exercise, get adequate sleep, and regulate emotions during this period. [3] HCG Trigger Day: Participants were informed of the HCG injection timing and precautions. They were encouraged to rest and relax after the injection. [4] Oocyte Retrieval Day: Pre-procedure instructions were provided. Post-retrieval care included recommendations for a light diet, high-calorie and high-protein meals, avoidance of strenuous exercise, sufficient sleep, medication adherence, and stress reduction. [5] Embryo Transfer Day: Participants were guided on pre-transfer preparations, such as maintaining a light diet. Normal activities were permitted with precautions against strenuous exercise. They were reminded to maintain a calm demeanor and seek medical attention promptly if abnormalities arose.

Participants in the experimental group received SFA interventions in addition to routine nursing care. The SFA protocol involved five sessions, each lasting 30–60 minutes, conducted on the following key dates: treatment initiation, first day of ovulation induction, HCG trigger day, oocyte retrieval day, and embryo transfer day. The intervention followed the five-step SFA process (Fig 1): [1] Problem Description: Participants were encouraged to describe the primary events contributing to their anxiety and depression. Normalization techniques were used to reassure participants that such concerns are common and valid. For example, advanced-age patients worried about ovulation outcomes were reassured that their concerns were shared by others in similar situations. [2] Goal Construction: Participants were guided to visualize the resolution of their issues and collaboratively set achievable action goals. Through the “miracle question” technique, participants were encouraged to describe their desired post-resolution state and establish actionable goals. For instance, they were prompted to imagine how they would feel and behave after experiencing successful treatment outcomes and restful sleep. [3] Exploration of Exceptions: Participants were assisted in identifying past successful coping strategies or positive experiences to address current challenges. Exception questions, such as “When your symptoms were less severe, what coping strategies worked?” helped participants rediscover effective methods like listening to music or engaging in physical activity, boosting their confidence and constructing new solutions. [4] Positive Feedback: Researchers provided dynamic feedback throughout the intervention to reinforce progress, enhance self-efficacy, and adjust intervention plans as needed. Participants were praised for their strengths and achievements, fostering a sense of accomplishment. For instance, if a participant reported reduced anxiety after learning about infertility treatments, they were commended for their proactive attitude. [5] Progress Evaluation: Participants’ progress was assessed using scaled questions, such as rating anxiety levels from 1 (most severe) to 10 (least severe). Progress evaluations guided the establishment of subsequent goals. Participants developed confidence in their ability to resolve issues independently as they observed their own progress.

### Measures

### General information questionnaire

The general information questionnaire, developed under the guidance of reproductive experts, captured the following: [1] Sociodemographic Information: Age, body mass index (BMI), family income, marital status, occupation, and educational level. [2] Disease-Related Variables: Causes and types of infertility, duration of infertility, number of pregnancies, and presence of biological children.

### Self-rating anxiety scale (SAS) [26]

The SAS evaluates subjective anxiety symptoms and consists of 20 items measuring psychological and physical symptoms. Participants responded based on their experiences in the past week, with item scores ranging from 1 to 4. The total raw score (20–80) was multiplied by 1.25 to yield a standard score. The SAS demonstrates excellent reliability (Cronbach’s α=0.88).

### Self-rating depression scale (SDS) [27]

The SDS measures depressive symptoms through 20 items scored on a 4-point Likert scale. Total scores (20–80) were multiplied by 1.25 to yield a standard score, with higher scores indicating more severe depression. The scale has strong reliability (Cronbach’s α = 0.791).

### Pittsburgh sleep quality index scale (PSQI) [28]

PSQI was used to evaluate the sleep quality in recent one month. The Chinese version of PSQI [29] was used to evaluate the sleep quality of patients with early threatened miscarriage after IVF-ET. PSQI has 18 items and 7 dimensions. Each dimension is evaluated by 0 ~  3 points, and the total score is 0 ~  21 points. The scale shows good internal consistency (Cronbach’s α= 0.73).

### Chinese version of reproductive quality of life scale (FertiQoL) [30]

The Chinese version of the Fertility Quality of Life (FertiQoL) scale is an internationally recognized tool designed to evaluate quality of life among individuals experiencing infertility. The questionnaire comprises 36 items, categorized into three main components: [1] Subjective Assessment: Includes two items (not scored) that assess overall quality of life and self-rated health status. [2] Core Module (Core-FertiQoL): Contains four domains—marital relationships, emotions, cognition and physical well-being, and social relationships. [3] Selective Treatment Module (Treatment-FertiQoL): Comprises two domains assessing the participant’s perception of the treatment environment and their tolerance of the treatment process. Each item is scored using a 5-point Likert scale ranging from 0 to 4, with a total score ranging from 0 to 100. Higher scores signify a better quality of life. The tool demonstrates excellent internal consistency, with Cronbach’s alpha coefficients ranging from 0.72 to 0.92 for both the total scale and sub-scales.

### Satisfaction questionnaire

A researcher-developed satisfaction questionnaire was employed to assess participants’ perceptions of the intervention. This tool consisted of five items, including the environment, content, number of interventions, total duration of intervention, and overall intervention satisfaction of the focused solution model (S1 Table).

### Data analysis

Epidata 3.1 software was used for data entry by two researchers. After double check, SPSS 25.0 software was used for statistical data analysis. The sociodemographic and disease characteristics were summarized as mean and SD, while categorical variables were expressed in frequency and percentage. The chi-square (χ2) test and t-test were used to compare values between two groups (Original data in S2 Table). Logistic regression was employed to analyze the relationship between intervention (or not) and anxiety, depression, sleep quality, quality of life, as well as clinical pregnancy rate. Alpha level was 0.05.

## Results

### Characteristics of the patients

The study included a total of 112 advanced age female patients undergoing IVF-ET, with 56 in the intervention group and 56 in the control group. In the intervention group, there were 4 cases lost to follow-up, including 2 cases lost after the second intervention and 2 cases with canceled transplantation, resulting in 52 cases with completed data collection. In the control group, there were 3 cases lost to follow-up, all due to canceled transplantation, resulting in 53 cases with completed data collection. The general characteristics of the patients who were lost to follow-up and those who completed the study were compared using the chi-square test and Fisher’s exact test, and no statistically significant differences were found (P > 0.05).

Ultimately, a total of 105 patients were included in the analysis, with a total effective rate of 93.75%. The age range was 35 to 48 years, with an average age of (41.12  ±  4.12) years. The distribution of BMI categories was as follows: BMI < 18.5 kg/m² accounted for 28.6%, 18.5 ~ 23.9 kg/m² accounted for 46.6%, and BMI ≥ 24 kg/m² accounted for 24.8%. Among the participants, 84.8% were of Han ethnicity, and 15.2% were from ethnic minorities. Based on the highest education level of the patients, 25.7% had completed junior high school or below, 39.0% had completed high school or vocational school, 32.4% had completed college or undergraduate studies, and 2.9% had completed graduate studies or above. Regarding the residence, 34.3% of the participants lived in rural areas, while 65.7% lived in urban areas. Among the study participants, 81.9% were employed. The majority of participants (53.3%) reported a monthly per capita family income of 5001 ~ 10000 yuan. In terms of marital status, 63.8% were first-time married women, and 36.2% were remarried. The duration of infertility was mostly 4 ~ 5 years (41.0%), and the treatment duration was 1 ~ 2 years, 3 ~ 4 years, and ≥ 5 years, accounting for 36.2%, 38.2%, and 28.6%, respectively. Among the participants, the leading cause of infertility was female-related (48.6%), while 39.0% already had biological children, and 61.0% had no biological children. The comparison of socio-demographic data between the two groups of patients using the chi-square test showed no statistically significant differences (P > 0.05) (Table 1).

**Table 1:** Baseline sociodemographic data and disease-related variables of two groups (n = 105).

Variables	SFA(n = 52)	CG(n = 53)	*χ* ^*2*^	*P*
Age			2.154	0.341
35 ~ 39	26(50.0%)	19(35.8%)		
40 ~ 44	15(28.8%)	20(37.7%)		
≥45	11(21.2)	14(26.4%)		
BMI(kg/m^2^)			0.760	0.684
<18.5	16(30.8%)	14(26.4%)		
18.5 ~ 23.9	25(48.1%)	24(45.3%)		
≥24	11(20.8%)	15(28.3%)		
Ethnicity			2.522	0.112
Han	47(90.4%)	42(79.2%)		
others	5(9.6%)	11(20.8%)		
Location			0.232	0.63
Rural	19(36.5%)	17(32.1%)		
Urban	33(63.5%)	36(67.9%)		
Education			3.343	0.342
Junior school and below	16(30.8%)	11(20.8%)		
Senior School	16(30.8%)	25(47.2%)		
Junior college or Bachelor’s degree	18(34.6%)	16(30.2%)		
Master’s degree and above	2(3.8%)	1(1.9%)		
Occupation			1.492	0.222
Unemployed	7(13.5%)	12(22.6%)		
Employed	45(86.5%)	41(77.4%)		
Family income (Yuan/monthly)			2.276	0.517
≤3000	3(5.8%)	3(5.7%)		
3001–5000	21(40.4%)	15(28.3%)		
5000–10000	24(46.2%)	32(60.4%)		
≥10000	4(7.7%)	3(5.7%)		
Marital status			0.111	0.739
First marriage	34(65.4%)	33(62.3%)		
Remarriage	18(34.6%)	20(37.7%)		
Presence or absence of biological children			0.077	0.781
Yes	21(40.4%)	20(37.7%)		
No	31(59.6%)	33(62.3%)		
Duration of infertility (years)			0.465	0.793
1 ~ 3	12(23.1%)	14(26.4%)		
4 ~ 5	23(44.2%)	20(37.7%)		
≥6	17(32.7%)	19(35.9%)		
Years of treatment (years)			0.163	0.922
1 ~ 2	18(34.6%)	20(37.7%)		
3 ~ 4	20(38.5%)	17(32.1%)		
≥5	14(26.9%)	16(30.2%)		
Causes for infertility			2.026	0.567
Male	10(19.2%)	7(13.2%)		
Female	27(51.9%)	24(45.3%)		
Both	8(15.4%)	12(22.6%)		
Unexplained	7(13.5%)	10(18.9%)		
Only-child			1.191	0.275
Yes	10(19.2%)	15(28.3%)		
No	42(80.8%)	38(71.7%)		

### Between-group comparison of SAS, SDS, PSQI, and FertiQoL scores before and after intervention

**Fig 2:**
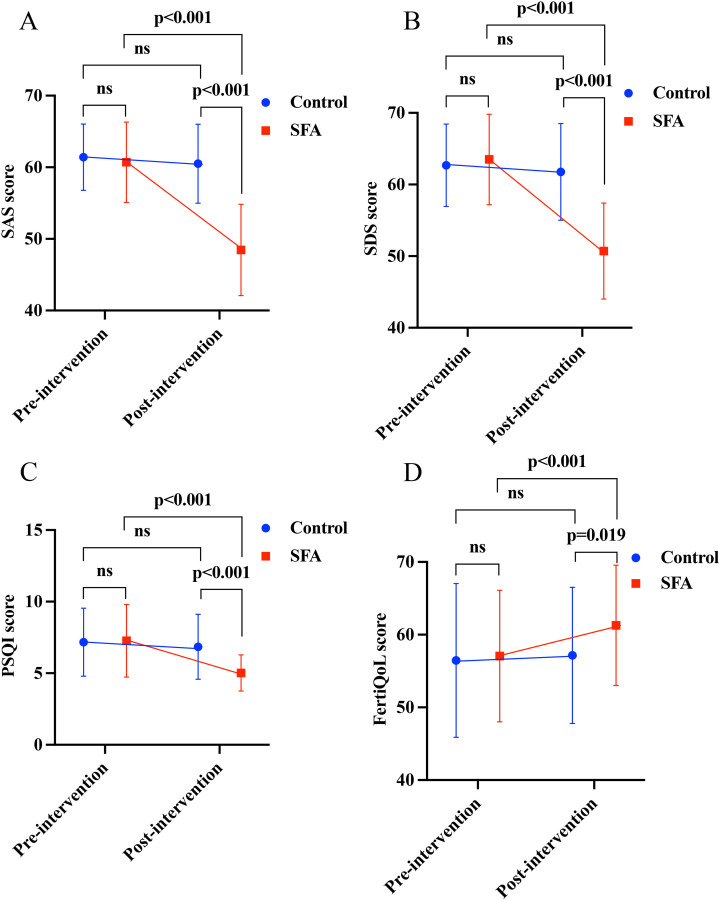
Analysis of SAS, SDS, PSQI, and FertiQoL scores. A analysis of SAS score in group control and SFA before and after intervention. **B** Analysis of SDS score in group control and SFA before and after intervention. **C** Analysis of PSQI score in group control and SFA before and after intervention. **D** Analysis of FertiQoL score in group control and SFA before and after intervention.

Before the intervention, there were no statistically significant differences in the SAS, SDS, PSQI, and FertiQoL scores between the two groups (P > 0.05). After the intervention, the SAS score of the intervention group was significantly lower than that of the control group (t = -10.348, P < 0.001); the SDS score of the intervention group was lower than that of the control group, with statistical significance (t = -8.416, P < 0.001); the PSQI score of the intervention group was lower than that of the control group (t = -5.087, P < 0.001); and the FertiQoL score of the intervention group was higher than that of the control group, with statistical significance (t = 2.389, P = 0.019) (Fig 2).

### Within-group comparison of SAS, SDS, PSQI, and FertiQoL scores before and after intervention

In the control group, there were no statistically significant differences in the SAS, SDS, PSQI, and FertiQoL scores before and after the intervention (P > 0.05). After the intervention in SFA group, the SAS score was significantly lower than before (t = 11.906, P < 0.001); the SDS score was lower than before (t = 14.991, P < 0.001); the PSQI score was lower than before, with statistical significance (t = 7.055, P < 0.001); and the FertiQoL score was higher than before (t = -2.828, P < 0.001) (Fig 2). Regression analysis was conducted, using intervention as the dependent variable (Table 2). The results indicated that SFA was correlated with lower anxiety and depression levels, lower sleep scores, and higher quality of life scores.

**Table 2:** Regression analysis of the relationship between intervention and anxiety, depression, sleep quality, quality of life, and clinical pregnancy rate.

Items	β	S.E	Z	*P*	OR (95%CI)
SAS Score	-0.30	0.06	-5.47	<0.001	0.74 (0.66 ~ 0.82)
SDS Score	-0.23	0.05	-5.18	<0.001	0.79 (0.72 ~ 0.86)
PQSI Score	-0.66	0.16	-4.19	<0.001	0.52 (0.38 ~ 0.70)
FertiQoL Score	0.06	0.02	2.26	0.024	1.06 (1.01 ~ 1.11)

### Clinical pregnancy rate and patient satisfaction after intervention

After the completion of the SFA intervention, follow-up was conducted until 28-30 days after transplantation to evaluate the pregnancy outcomes of the patients. In the intervention group, there were 25 cases of clinical pregnancy (48.1%), and in the control group, there were 24 cases of clinical pregnancy (45.3%). The clinical pregnancy rate did not show a statistically significant difference between the two groups (Table 3).

**Table 3:** Between-group comparison of clinical pregnancy rate after intervention(%).

Items	n	clinical pregnancy(n,%)	*χ* ^*2*^	*p*
SFA group	52	25(48.1%)	0.082	0.774
Control group	53	24(45.3%)		

In the intervention group, a total of 52 advanced age IVF-ET female patients were included, and they showed high satisfaction with the intervention content and environment. However, two patients expressed dissatisfaction with the number and total duration of interventions (Table 4).

**Table 4:** Patient satisfaction with SFA intervention(%).

Items	Very satisfied(%)	Satisfied(%)	Dissatisfied(%)
Intervention content	33(63.5%)	19(36.5%)	0
Intervention environment	37(71.2%)	15(28.8%)	0
Intervention frequency	25(48.1%)	25(48.1%)	2(3.8%)
Total duration of intervention	34(65.4%)	16(30.8%)	2(3.8%)
Overall satisfaction	20(38.5%)	32(61.5%)	0

## Discussion

The present study included 105 advanced age female patients undergoing IVF-ET, with pre-intervention SAS scores of (60.70 ± 5.61) and SDS scores of (63.51 ± 6.31), indicating moderate levels of anxiety and depression. The results of the study showed that SFA significantly improved anxiety, depression, and sleep quality in advanced age infertile women, and it also improved their fertility-related quality of life.

Age and ovarian reserve are important indicators affecting the outcome of assisted reproductive technologies [31,32]. As age increases, female ovarian function declines, leading to reduced response to ovulation-inducing medications, resulting in fewer follicles and decreased egg quality, which in turn affects the embryo implantation rate [33]. Advanced age infertile women belong to a group with lower success rates in infertility treatment, as data from the Centers for Disease Control and Prevention (CDC) shows that the success rate of IVF-assisted pregnancy decreases linearly with age after 35 years old [34]. Throughout the treatment process, issues such as medication-induced ovulation, monitoring of reproductive hormone levels in the blood, repeated invasive transvaginal ultrasounds, fear of surgical pain, and concerns about the quality of embryos create a constant state of anxiety and depression in patients [35]. SFA can effectively improve the anxiety levels of advanced age women undergoing IVF-ET, and its intervention effect is superior to that of conventional nursing. The core of the intervention is to provide goal-directed problem-solving methods, gradually guiding patients to achieve goals by tapping into their potential and utilizing available resources, thereby improving their negative emotions [21]. SFA encourages patients to describe their problems, facilitating cognitive reconstruction, guiding patients to approach external events with positive behavior, enhancing their sense of self-control over these events, and strengthening their confidence in overcoming the disease, ultimately achieving the goal of improving anxiety [36]. The present study used the five stages of SFA to help patients face their problems, establish goals, explore solutions, establish positive behaviors, provide timely feedback and adjustment, encourage continuous progress, and ultimately break free from negative emotions. The results of this study are consistent with those of Huang C et al. [36] and Song Y et al. [37], who found that the use of SFA effectively reduces patients’ negative thought patterns, improves their emotion-regulation abilities, and ultimately relieves anxiety and depression. Through practicing SFA, patients can transform their beliefs and cognitive patterns, promoting self-adjustment and coping. Compared to traditional knowledge transmission, SFA enhances patient initiative and participation while fully respecting patients [37].

Sleep plays a significant role in metabolism and reproductive function [38]. When patients experience severe negative emotions, they may encounter difficulties falling asleep, insomnia, and early morning awakening; sleep disorders can affect memory, judgment, and lead to nervousness, and in severe cases, cause anxiety and depression [39]. Sleep disorders may result in prolonged conception periods, reduced chances of pregnancy, and increased miscarriage rates in women [40]. This study found that advanced age infertile patients had significant sleep-related problems, including erroneous beliefs about sleep, unrealistic expectations, erroneous attributions of insomnia, and exaggeration of the consequences of insomnia. Research shows that irrational sleep beliefs are one of the key psychological factors affecting sleep quality. Unrealistic attitudes and opinions about sleep create anticipatory anxiety in patients, enhancing their physiological and psychological arousal levels, ultimately leading to a vicious cycle of sleep problems [41]. In this study, through inspired guidance, patients were encouraged to capture positive resources, advantages, and successful experiences from their stories, avoid excessive thinking about the consequences of insufficient sleep, and prevent self-imposed pressure to fall asleep. During the intervention, patients were guided to appropriately reduce daytime sleep to ensure sleep quality. On the other hand, after the positive influence and impact of the SFA on patients’ depression and anxiety emotions, it can break the vicious cycle of anxiety-insomnia-anxiety, affect the patient’s cortical function, stimulate the release and secretion of neurotransmitters such as dopamine and norepinephrine, reduce their worries about insomnia symptoms, and consequently improve sleep quality [42,43]. In this study, the SFA is a process of helping patients to reconstruct while simultaneously being ability-oriented, resilience-oriented, and motivation-enhancing. This promotes patients to improve their emotional management capabilities, break free from the distress of negative emotions such as anxiety and depression, and thereby improve sleep quality.

In this study, the fertility-related quality of life of advanced age women undergoing IVF-ET was lower than that of women with infertility in the reproductive age group [44]. SFA played a positively facilitating role in improving the fertility-related quality of life in advanced age women undergoing IVF-ET. Depression can lead to a reduction in patients’ quality of life and even adversely affect treatment outcomes [45]. Therefore, during the intervention process, listening to the patients’ narratives provides them with the opportunity to vent their emotions and helps them clarify their problems. Simultaneously, providing explanations of IVF-ET treatment-related knowledge, using successful cases as examples, and avoiding negative rumination that repeatedly worries the patients increase their confidence in the treatment. SFA can gradually enhance the patient’s quality of life by addressing their psychological distress [46].

In this study, the SFA did not significantly increase the clinical pregnancy rate in advanced age women undergoing IVF-ET, which is consistent with other research results [47,48]. Relevant meta-analysis results suggest that psychological interventions have limited impact on infertility pregnancy outcomes [49]. The pregnancy outcomes of advanced age women are influenced by various factors, including the number of IVF cycles, baseline FSH levels, endometrial thickness on the day of HCG injection, number of retrieved oocytes, etc. [50,51]. In our study, the positive impact on sleep quality, emotional well-being, and quality of life, which are crucial for the overall health of patients undergoing IVF-ET. These improvements are meaningful, as reducing stress and enhancing quality of life may indirectly support treatment adherence and overall patient health, even if they do not directly impact pregnancy rates.

Patients in this study showed a generally high overall satisfaction with the SFA. Two patients expressed dissatisfaction with the number and duration of the interventions. Research indicates that SFA can achieve significant effects with 3 to 5 interventions. Compared to other methods, the frequency and duration of interventions are not crucial. The key is to prompt patients to discover exceptional experiences in their personal perceptions, focus on current goals, and explore future possibilities, gradually forming their own solutions and construction [52]. Therefore, in the implementation of subsequent intervention plans, emphasis should be placed on improving patients’ ability to identify problems using their own strengths, stimulate their potential to envision achievable goals, and gradually achieve them.

SFA, as a non-pharmacological intervention, can be implemented with minimal cost and without additional medical resources. Unlike pharmacological treatments, which may have side effects or contraindications, SFA offers a safe alternative to support the emotional well-being of patients. The approach is well-suited for integration into existing workflows without extensive additional training for healthcare professionals, making it a feasible option for resource-limited settings. The incorporation of SFA could help create a more supportive environment, which ultimately contributes to a more positive patient experience.

Future psychological intervention research is suggested to incorporate physiological and biochemical indicators into outcome measures, such as psychological stress markers: catecholamines, cortisol, salivary alpha-amylase, etc., to further validate the improvement effect of psychological interventions on patients’ psychological conditions through monitoring changes in psychological stress marker levels. Longitudinal studies can be conducted to explore the psychological characteristics and major psychological issues of patients at different stages of IVF-ET treatment, identifying the optimal time for psychological intervention to achieve better psychological treatment outcomes. In addition, researchers can fully utilize the internet to expand the application of psychological interventions, actively conduct online practical courses, and provide patients with a more straightforward and easily accessible intervention platform.

There are some limitations in this research. The sample size of this study is from a single hospital. This study uses self-reported scales to measure patients’ psychological status, which may be at risk of overestimation and social desirability bias in the research results. Due to limitations in human and material resources, as well as the high patient turnover, long-term follow-up evaluations were not conducted after patient discharge. This study cannot determine the long-term impact of SFA on patients’ emotional management.

## Conclusion

This study confirms the positive effects of SFA on anxiety and depression in advanced age women undergoing IVF-ET, as well as its improvement on sleep quality and quality of life. The study also validates the effectiveness and applicability of the intervention plan in psychological nursing for advanced age infertile women. The results of this study may contribute to the construction of a psychological intervention plan for advanced age women undergoing IVF-ET.

Assisted reproductive technology nurses can use SFA to solve problems such as psychological distress and sleep in advanced age women. SFA adopts a problem-oriented model, with clear intervention goals. It leads directly to available resources or experiences through multiple complex problem events of the research subjects, continuously achieving expected objectives based on the subjects’ potential. SFA has high feasibility in clinical practice, patients can learn and practice by themselves, the learning cost is low, and it has practical operability and effectiveness.

## Supporting information

S1 Table Satisfaction questionnaire. (DOCX)

S2 Table Group SFA-before intervention. (XLSX)

S3 Table Group SFA-after intervention. (XLSX)

S4 Table Group control-before intervention. (XLSX)

S5 Table Group control-after intervention. (XLSX)

S6 Table Lost cases. (XLSX)

S7 Table Pre-Experiment. (XLSX)

## Abbreviations

SFA: Solution-focused approach
IVF-ET: In vitro fertilization and embryo transfer
ART: Assisted Reproductive Technology
HPA: Hypothalamic-Pituitary-Adrenal axis
HPGA: Hypothalamus-Pituitary-Gonad Axis
SAS: Self-rating anxiety scale
SDS: Self-rating depression scale
PSQI: Pittsburgh sleep quality index scale
FertiQoL: Chinese version of reproductive quality of life scale
