# Supplementary material for: Effect of solution focused approach on women aged 35 or over with in vitro fertilization-embryo transfer: A quasi-experimental trial
Source: PLoS One. 2025 Mar 19;20(3):e0316771. doi: 10.1371/journal.pone.0316771 (PMC11922246; doi:10.1371/journal.pone.0316771)
Supplement: S1 Table — (DOCX) [file pone.0316771.s001.docx]

| Supplementary table 1 Satisfaction questionnaire | |
| --- | --- |
| Question | Answer |
| Are you satisfied with the content of our SFA intervention? | □Very satisfied □Satisfied □Uncertain □Dissatisfied □Very dissatisfied |
| Are you satisfied with the environment for SFA interventions? | □Very satisfied □Satisfied □Uncertain □Dissatisfied □Very dissatisfied |
| Are you satisfied with the number of interventions during the SFA? | □Very satisfied □Satisfied □Uncertain □Dissatisfied □Very dissatisfied |
| Are you satisfied with the total duration of implemented SFA during this treatment cycle? | □Very satisfied □Satisfied □Uncertain □Dissatisfied □Very dissatisfied |
| What is your overall satisfaction with our SFA intervention? | □Very satisfied □Satisfied □Uncertain □Dissatisfied □Very dissatisfied |
